# Diagnosis of manganism and manganese neurotoxicity: A workshop report

**DOI:** 10.3892/mi.2024.135

**Published:** 2024-02-06

**Authors:** Donald R. Mattison, Franco Momoli, Cemil Alyanak, Michael Aschner, Marissa Baker, Neil Cashman, Ulrike Dydak, Nawal Farhat, Tomás R. Guilarte, Nataliya Karyakina, Siva Ramoju, Natalia Shilnikova, Pille Taba, Daniel Krewski

**Affiliations:** 1 Risk Sciences International, Ottawa, ON K1P 5J6, Canada; 2 School of Epidemiology and Public Health, University of Ottawa, Ottawa, ON K1G 5Z3, Canada; 3 Arnold School of Public Health, University of South Carolina, Columbia, SC 29208, USA; 4 Department of Molecular Pharmacology, Albert Einstein College of Medicine, Bronx, NY 10461, USA; 5 Department of Environmental and Occupational Health Sciences, University of Washington, Seattle, WA 98195, USA; 6 Djavad Mowafaghian Centre for Brain Health, University of British Columbia, Vancouver, BC V6T 1Z3, Canada; 7 ProMIS Neurosciences, Inc., Toronto, ON M4S 3E2, Canada; 8 School of Health Sciences, Purdue University, West Lafayette, IN 47907, USA; 9 Department of Environmental Health Sciences, Robert Stempel College of Public Health and Social Work, Florida International University, Miami, FL 33199, USA; 10 R. Samuel McLaughlin Centre for Population Health Risk Assessment, University of Ottawa, Ottawa, ON K1G 5Z3, Canada; 11 Department of Neurology and Neurosurgery, Institute of Clinical Medicine, University of Tartu, 50406 Tartu, Estonia; 12 Neurology Clinic, Tartu University Hospital, 50406 Tartu, Estonia

**Keywords:** manganese, manganism, diagnostic criteria, manganese neurotoxicity, Parkinson's disease

## Abstract

With declining exposures to manganese (Mn) in occupational settings, there is a need for more sensitive exposure assessments and clinical diagnostic criteria for manganism and Mn neurotoxicity. To address this issue, a workshop was held on November 12-13, 2020, with international experts on Mn toxicity. The workshop discussions focused on the history of the diagnostic criteria for manganism, including those developed by the Institut de Recherche Robert-Sauvé en Santé et en Sécurité du Travail (IRSST) in Quebec in 2005 and criteria developed by the Chinese government in 2002 and updated in 2006; the utility of biomarkers of exposure; recent developments in magnetic resonance imaging (MRI) for assessing Mn accumulation in the brain and diagnosing manganism; and potential future applications of metabolomics. The suggestions of the participants for updating manganism diagnostic criteria included the consideration of: i) A history of previous occupational and environmental exposure to Mn; ii) relevant clinical symptoms such as dystonia; iii) MRI imaging to document Mn accumulation in the neural tissues, including the basal ganglia; and iv) criteria for the differential diagnosis of manganism and other neurological conditions. Important research gaps include the characterization of Mn exposure and other co-exposures, exploration of the roles of different brain regions with MRI, understanding the complexity of metal ion transporters involved in Mn homeostasis, and a need for information on other neurotransmitter systems and brain regions underlying the pathophysiology of manganism.

## Introduction

Manganism, first identified by James Couper in 1837, is a neurotoxic condition resulting from chronic exposure to manganese (Mn) (1). It results from the inhalation of low soluble Mn dust or fumes at levels >1 mg Mn/m^3^ of respirable Mn in total dust in industrial settings, including mining, metallurgical operations and welding. Manganism may also be a result of exposure by other routes (e.g., intravenous injections in drug addicts or total parenteral nutrition) (2,3), or due to genetic mutations causing manganese metabolic disorders (4-7). For decades, manganism has been associated with high levels of exposure to Mn in occupational settings. Mn exposure levels among workers as high as 926,000 µg/m^3^ in the 1950s have been described in the literature (8). Given that Mn exposure is far lower than it was historically, manganism from chronic Mn exposure is rarely expected from current working conditions.

While long recognized as a progressive and serious debilitating illness, manganism remains imprecisely defined in clinical terms and is difficult to diagnose, particularly in its early phases. Prior attempts that have developed operational definitions of manganism are based on classic characteristics of the disease associated with historic high exposures to Mn rather than levels of modern exposure (9). There is a need for a more sensitive clinical protocol and exposure assessment for manganism, and the need is becoming more pressing as declining exposures to Mn lead to more subtle symptoms. A number of factors warrant consideration for a modern diagnosis of manganism or manganese-toxicity: i) The most recent proposed diagnostic criteria were published in 2006 and could benefit from an update, including modern imaging and biomarker evidence; ii) there is no general agreement on the most reliable tests for detecting changes in neurological signs and/or symptoms, including subtle changes, in subjects exposed to Mn; iii) degrees of certainty associated with specific tests or combinations of tests in diagnosing of manganism are not documented; and iv) in diagnosing manganism, accurate information on exposure to Mn is not always available.

To gain clarity on key elements that should be considered in the refinement of currently available definitions for manganism, a virtual workshop on diagnostic criteria was held on November 12-13, 2020 on Zoom (the workshop agenda is presented in Appendix S1). The workshop sought advice from international experts on Mn toxicity on the characterization of manganism, the differentiation of manganism from other degenerative conditions, and the key criteria that should be considered-given changes in occupational Mn exposure - in the diagnosis of manganism today. Participants were charged to provide advice on the key criteria considerations involved in the diagnosis of manganism and Mn toxicity. Specifically, the panel was asked to consider more recent updates on the use of biomarkers of exposure, imaging markers, distinguishing features from Parkinson's disease (PD) and other motor neuron diseases, and more recent evidence from scientific studies.

In this report, an overview of the current knowledge regarding Mn toxicology is provided and the main issues discussed and suggested by the workshop participants on the diagnosis of manganism considering recent developments are summarized.

## Current diagnostic criteria for manganism

Manganism is a specific central nervous system syndrome caused by high levels of exposure to Mn. It tends to be heterogeneous in its presentation, course and outcome, and does not have a single clinical, laboratory, pathological, or neuroimaging feature that could serve as a ‘gold standard’ in support of its diagnosis. Diagnostic criteria have been proposed for both research purposes and for occupational health. For example, Myers *et al* (10) described a ‘cascaded screening process’ to estimate the prevalence of clinical manganism in a South African smelter; using nine questions and nine neurological tests, more careful review would be triggered by the presence of more than one symptom (e.g., an irritated mood, difficulty concentrating), a neurological sign (e.g., unbalanced toe-heel walking, hand tremor), or blood Mn levels >40 µg/l. Two commonly cited occupational health diagnostic procedures are described below.

### Institut de Recherche Robert-Sauvé en Santé et en Sécurité du Travail (IRSST) proposed diagnostic criteria

The IRSST developed guidance on the diagnosis of manganism in the workplace established by international clinical experts (11). Mn-exposed workers are classified into three groups based on the level of diagnostic certainty in view of the integrity of diagnostic findings, as well as the sensitivity and specificity of the diagnostic tools. Diagnostic findings used to identify cases are derived from a range of sources including exposure data, clinical findings, and pharmacological, neuro-imaging and histopathological tests. The following guidelines are provided by the IRSST:

i) A diagnosis of clinically *possible* case of occupational manganism if the following three criteria are met: a) there must be a documented identifiable source of occupational Mn exposure; b) at least one neurological symptom among the symptoms: tremor, bradykinesia, rigidity and postural instability; and c) symptoms and clinical signs of neuropsychological disturbances, mainly motor disturbances.

ii) A diagnosis of clinically *probable* manganism requires all conditions from a possible case of manganism plus a) evidence of neuropsychological disturbances related to basal ganglia; b) absence of or unsustained pharmacological response to levodopa; and c) exclusion of other neuropsychological diseases related to basal ganglia, such as PD.

iii) A diagnosis of *definite* manganism requires that a clinically probable case has a) histopathological confirmation obtained from an autopsy (if available) or a cerebral biopsy (if available); and b) a normal Fluoro-Dopa or dopamine transporter (DAT) positron emission tomography (F-Dopa PET or DAT PET) scan (a normal scan would confirm manganism, but an abnormal scan would not exclude the disease).

### Ministry of Health (China) diagnostic criteria of occupational chronic manganism

Criteria for diagnosing manganism were also developed by the Ministry of Health of the People's Republic of China in 1982 and revised in 2002 and 2006 (for inclusion of moderate poisoning). The 2006 criteria (12) provide diagnostic considerations for three levels of Mn poisoning and are summarized as follows based on an internal translation for the purpose of this commentary:

i) Assessment of occupational exposure history and clinical manifestations (mainly extrapyramidal tract damage) in addition to information obtained from industrial air monitoring and personal spot air Mn concentrations.

ii) Distinguishing chronic Mn poisoning from diseases such as PD, hepatolenticular degeneration (Wilson's disease), acute carbon monoxide poisoning, delayed encephalopathy, senile tremor, cerebral arteriosclerosis and mental illness.

iii) Subjective symptoms: Dizziness, headache, fatigue, dyssomnia, forgetfulness, autonomic nervous system disorders (loss of appetite, salivation, sweating, palpitations, loss of libido) and possibly, limb pain with weakness and heaviness in the lower limbs.

iv) Together with the symptoms aforementioned, three levels of poisoning can be classified as follows: a) Mild poisoning: Increased muscle tone or tremor, finger tremors, depression, attention deficit, reduced interest in surroundings, or irritability and hyperactivity, as well as changes in speech; b) moderate poisoning: In addition to the characteristics of mild poisoning, there is an increase in the muscle tone of the limbs, often accompanied by resting tremor; c) severe poisoning: In addition to the characteristics of moderate poisoning, individuals with one of the following conditions can be diagnosed with severe poisoning:

*Obvious extrapyramidal damage*: A significant increase in muscle tone throughout the body; tremor in limbs; tremors may affect the jaw, head, and neck; abnormal gait.

*Severe mental disorders*: significant mental and emotional changes such as apathy, slow reaction, involuntary crying and laughter, obsessive-compulsive ideas, impulsive behavior, and intellectual disabilities.

v) Mn concentration in the urine or hair [this is in the earlier 2002 criteria, as described by Rutchik *et al* (13)].

Manganism and PD exhibit some similarities, notably the presence of generalized bradykinesia and widespread rigidity (14). Manganism and PD affect different areas of the brain, which allows for a distinction between the two syndromes based on symptoms (Table I). The substantia nigra pars compacta dopaminergic neurons are progressively lost in PD, whereas neuronal loss and gliosis in the globus pallidus, the substantia nigra pars reticulata and the striatum predominately characterize manganism at the morphological level (15). Manganism is characterized by features not found in PD, such as less-frequent resting tremor, more frequent dystonia, propensity to fall backward, no response to levodopa treatment, and no detection of a reduction in fluorodopa uptake by PET scan (14) and DaT/SPECT scan (16). A flow diagram of neuroimaging considerations is provided in Fig. S1 [adopted from Kim (17)]. Lewy body formation-not observed in manganism - is a critical diagnostic criterion of PD, and could be confirmed by autopsy. The differentiation of clinical signs and imaging of manganism from PD should not pose any problem for experienced clinicians (18).

## Summary of workshop discussions

### Biomarkers of exposure

Several authors of the present study have recently published detailed critical reviews of occupational (20) and environmental (21) biomarkers of Mn exposure. Although certain serum biomarkers of Mn exposure are promising, workshop participants generally agreed that no ideal biomarkers are currently available and that different biomarkers represent different windows of exposure. In addition, participants stressed the need for standardized tests for Mn in different biological media for comparability across studies.

Mn in hair and toenails as potential biomarkers were discussed. A previous study conducted by participants at Albert Einstein College and Purdue University, examined the association between Mn exposure and Mn levels in hair (22). The findings of that study suggested no association between internal Mn dose and Mn accumulation in mouse or rat hair over a period of 60 days. These results do not support the use of Mn in hair as a valid biomarker for internal exposure to Mn at a neurotoxic level (22).

Data from another study suggesting promising signs of Mn in toenails as a biomarker of exposure from welders and controls (23) were presented. The findings indicated a strong linear correlation with previous exposure (7-12 months) in adults. In toenails, which are easy to collect, Mn exhibited a specificity of 91% and a sensitivity of 94% in discriminating between exposed and unexposed subjects. Additional results presented suggest a significant correlation between toenail Mn and motor function (pegboard and finger tapping), but not with cognitive function (24).

Previous studies have discussed evidence on the association between inhalation exposure to Mn in occupational settings and Mn concentrations in biological media and tissues (25-28). The findings on the association between external Mn exposure indices and biological indicators of exposure were inconsistent, and suggest that at current occupational exposure levels, the blood Mn level is not a reliable biological indicator of long-term Mn exposure and related adverse health effects in workers exposed to Mn-containing dust or fumes.

Only a limited number of reviews have focused on the biomarkers of environmental Mn exposure. While two reviews (29,30) suggest that Mn in hair and nails may be promising as non-invasive biomarkers of Mn exposure, further research is required on the associations between Mn in environmental media and biological matrices.

The workshop participants discussed the challenges of using biological indicators and the lack of supporting evidence for their use for clinical purposes in occupational and non-occupational settings. It was noted that some studies have provided support for the use of Mn in toenails as a biomarker for motor function. In addition, plasma may have limited potential in subjects with higher or longer-term exposure (31,32).

### Magnetic resonance imaging (MRI)

There have been advances in the use of MRI for quantifying Mn accumulation in specific parts of the brain. Recent studies from welders have provided evidence that the accumulation of Mn in the human brain is not restricted to the basal ganglia. Significant accumulations have been reported in the frontal cortex, frontal white matter and the hippocampus (33,34) and whole-brain analysis has revealed elevated Mn particularly along white matter tracts (35). Furthermore, uptake and clearance vary according to the region of the brain (33). There was consensus among the workshop participants that MRI is useful as a short-term indication of exposure, since Mn washes out from certain parts of the brain after cessation of exposure.

It was also discussed that regions with highest hyperintensities on MRI are not necessarily those that are more closely associated with symptoms, exposure or pathological outcomes. As such, the focus should not only be on the globus pallidus, but should consider frontal white matter, the frontal cortex, substantia nigra and other hindbrain regions. One of the experts presented findings for seven welders followed over a 2-year period with decreasing exposure. The most notable findings were: i) Gamma-aminobutyric acid (GABA) levels in the thalamus decreased with decreasing exposure; ii) the globus pallidus R1 did not change significantly; iii) substantia nigra R1 decreased with decreasing exposure; and iv) frontal cortex R1 (unexpectedly) increased with decreasing exposures (33).

Participants discussed the use of MRI parameters as indicators of short-term Mn exposure, and how imaging data may be used in the diagnosis of manganism. T1-weighted MRI scans performed in occupationally exposed individuals have revealed widespread Mn deposition in the brain and accumulation of Mn within and outside the basal ganglia (36,37). Some studies have suggested that T1 signal intensities in brain areas other than the globus pallidus, particularly in the extra-pallidal basal ganglia, may reflect Mn exposure equally well or better than signal intensities in the globus pallidus (38,39). Further evaluation of potential applications of MRI imaging techniques in characterizing Mn concentrations in neural tissue is provided in a recent critical review by Jensen *et al* (40).

### Exposure considerations

Workshop participants stressed the need for improvements in Mn exposure assessment in studies on Mn toxicity, considering the range of exposure circumstances encountered in occupational settings. Consideration should also be given to exposure in toxic non-occupational conditions (e.g., hepatic or renal insufficiency). Ideally, exposure data should be collected using personal air sampling measurements within the breathing zone. This is critical for studies that aim to validate biomarkers of exposure.

In addition, the composition of welding fumes in terms of heavy metals (e.g., mercury, iron, nickel) should be considered and their impact should be elucidated. The need for careful characterization of occupational exposure circumstances involving mixed exposures to manganese and other metals was noted.

### Metabolomics

Metabolomics is broadly defined as the comprehensive assessment of metabolites and low-molecular-weight molecules in a biological sample (41). Utilizing mass spectrometry (MS) or nuclear magnetic resonance (NMR), this method allows for the profiling of potentially thousands of metabolites. Metabolomics can be undertaken as an exploratory analysis method to generate hypotheses between exposure or disease status and putative biomarkers or to confirm the association between annotated metabolites or pathways of metabolites and an exposure or disease.

Metabolomics as an alternative to traditional biomonitoring for Mn exposure has begun to be explored in the literature and shows future promise for identifying biomarkers of Mn exposure and understanding pathways in the body through which Mn exerts toxicity (42-44). In particular, metabolomics holds promise for Mn, given the absence of strong support for useful traditional biomarkers of Mn exposures, such as Mn levels in blood, plasma or urine (31,45). One of the workshop participants presented a study on 59 metabolites identified by NMR and 224 metabolites identified by MS in urine. The findings indicated differences in relative abundances of metabolites, and the pathways in which they operate, between Mn-exposed and unexposed workers (43). Although the results need to be interpreted with caution given the variability of metabolites due to internal and external cues, and the hypothesis-generation nature of the analysis, metabolomics and other emerging-*omics* technologies provide an opportunity for exploring the association between exposure and health outcomes and determining new biomarkers of exposure.

### Classical manganism

Manganism can develop as a result of genetic factors or exposure to Mn in occupational settings, including liver cirrhosis, total parenteral nutrition, genetic disorders related to Mn metabolism and drug abuse. Manganism can result from the inhalation of low soluble Mn dust or fumes at levels >1 mg Mn/m^3^ of respirable Mn in total dust in industrial settings (46). Chronic poisoning by Mn following long-term occupational exposure to high concentrations of low soluble Mn compounds results in accumulation of Mn within the basal ganglia, leading to manganism (47). The early onset of Mn intoxication is usually subtle and progressive. Initial signs can include non-specific non-motor neurological manifestations, psychiatric symptoms and extrapyramidal signs. Exposed individuals may complain of asthenia, anorexia, apathy, insomnia or drowsiness, malaise, somnolence, or a diminished libido or impotence. Psychiatric symptoms are more specifically indicative of Mn toxicity, including disorientation, emotional instability, compulsive acts, hallucinations, illusions, delusions, and slurring and stuttering speech with a diminished voice. These are followed by selective extrapyramidal disorders, such as an imbalance in walking or on arising, finger discoordination, dystonia and action tremor (46).

Numerous cases of manganism reported involve very high exposure levels in the mining industry. The literature describes exposure levels among South African miners between 190-780 µg/m^3^ of Mn (8-h TWA) (48) and as high as 926,000 µg/m^3^ among Moroccan miners (49).

Occupational exposure to Mn dust and welding fumes is currently several orders of magnitude less than levels associated with previously described cases of manganism. As such, the workshop participants agreed that occupational manganism is extremely rare today. However, very high levels of Mn can still be observed in individuals carrying genetic mutations [such as solute carrier family (SLC)30A10 and SLC39A14] (6,50,51) or in methcathinone/ephedrone users (3,52,53). Hence, the need to refine the definition of manganism.

Furthermore, hypermanganesemia, a disorder in which Mn accumulates in the body, may occur due to inherited mutations in the SLC30A10 and SLC39A14 genes encoding transporters involved in Mn homeostasis (54). There have been reports of MRI appearances of hyperintensities in the basal ganglia and white matter among patients not exposed to high Mn levels, but had a mutation in the SLC30A10 gene; the mutation may impair Mn elimination and induce hypermanganesemia causing Mn deposits in neural tissue (55).

## Key elements of diagnostic criteria for manganism

The workshop participants considered existing diagnostic criteria by IRSST (11) and the Ministry of Health of the People's Republic of China (12).

IRSST identified three categories of diagnostic criteria: The source of occupational exposure, neurological symptoms of motor dysfunction and clinical signs of neuropsychological disturbances. Although the IRSST criteria may still be applicable today, certain elements need to be specified.

Manganism is associated with high exposures to Mn or metabolic disorders that induce hypermanganesemia, resulting in Mn deposition in the basal ganglia and other brain regions. Lower exposures would not be expected to produce the same clinical syndrome. However, exposure to lower Mn levels could produce a more subtle neurotoxic response, that may lead to manganism or manganese toxicity under continued Mn exposure. It may be useful to consider manganism not only as an occupational disease, but as a disease associated with genetic or metabolic disorders or with excess exposure to Mn.

There was an extensive discussion around key elements to be considered for updated diagnostic criteria. In particular, the experts were requested to list the two most important elements to be included when updating the criteria. The most common element suggested was a history of exposure or the known origin of Mn burden. This would apply in cases of exposure in occupations with known exposure to Mn, as well as in non-occupational settings (for example, genetic mutations or methcathinone/ephedrone abuse).

Having symptoms also characteristic of parkinsonian clinical syndrome was another element indicated by the majority of the panel, although clinical manifestations should be characterized by clinicians. Of note, one expert suggested issues with at least one of the motor skills as an important element to consider.

Given that imaging techniques have advanced significantly since the publication of the IRSST criteria in 2005, the experts agreed that MRI confirmation of Mn accumulation in the basal ganglia (or brain) would aid in the diagnosis of manganism, however only in cases of recent Mn exposure. This corresponds to confirmation of hyperintensities in the basal ganglia in individuals that have been recently exposed to Mn.

The workshop participants suggested the inclusion of distinguishing features of manganism from other neurological conditions with similar symptoms in the updated criteria. This would aid in establishing a set of logical steps to guide diagnosis of manganism.

Regarding biomarkers included in the diagnostic criteria, there was an agreement that, currently, there is no ideal biomarker useful in diagnosis, although there is literature available supporting Mn in toenails as a useful biomarker to establish the fact of exposure over the previous 1 year.

## Research directions

Although numerous advancements have been achieved in research on Mn metabolism and pharmacokinetics, biomarkers of exposure and brain imaging, there is a need for further research to clarify the complex systems surrounding Mn in the human body. A number of gaps were identified during the workshop, namely the following:

### Better characterization of the roles of different brain regions

MRI research on typically exposed welders in the USA revealed that Mn deposition in the brains of welders, measured by the MRI longitudinal relaxation rate R1, is widespread and follows white matter tracts, likely contributing to the distribution of excess Mn throughout the brain (35).

The literature further indicates that Mn deposition exhibits a non-linear association with exposure (56), and that the association of R1 levels with the Mn exposure time windows varies for different brain regions (37), suggesting that different areas may be affected by Mn deposition sequentially.

Finally, a recent study demonstrated that different brain regions exhibit different elimination rates of Mn upon cessation of exposure (33). In particular, the frontal cortex may play a vulnerable role (34) and has shown increased Mn accumulation even upon cessation of exposure to Mn (33). Furthermore, Mn accumulation in the globus pallidus, typically the most prominent brain region accumulating Mn, has not always been found to be associated with cognitive or motor symptoms (37,57).

This demonstrates a gap in knowledge on the role and timing of Mn deposition in different brain regions in the understanding of Mn toxicity in occupational and environmental settings. There is a clear need for systematic studies on the spatio-temporal dynamics and dose-response relationship of uptake and elimination of brain Mn in humans on a whole-brain basis.

### Improvement of exposure characterization

Given the limitations of novel and traditional biomarkers of exposure to Mn, traditional exposure assessment of Mn exposure is still critical for characterizing exposed from unexposed, as well as their level of exposure.

In occupational cohorts, utilizing full-shift personal air sampling for inhalable [Institute of Occupational Medicine (IOM)] samplers, respirable (cyclones) or total dust fractions is considered the gold-standard. Ideally, each worker is sampled over multiple workdays to assess variability in daily exposure. If feasible, other information should be collected concurrently with personal airborne Mn exposure assessment, such as whether respiratory protection was used (this can influence the dose of Mn received), historical exposure to Mn (through collecting measures on job and task history) and confounding sources of exposure to Mn, such as through diet.

As particle size is a key determinant of metal fume deposition in the respiratory tract (58,59), assessing the size distribution of exposures may be another strategy with which to better characterize exposure and understand the association between exposure, dose and toxicity. When personal sampling is not feasible, exposure questionnaires should be developed that consider job tasks, frequency of tasks, the use of respiratory protection, detailed job history and outside sources of Mn exposure. This will allow for more accurate exposure modelling (60).

The community exposure assessment of Mn can be completed utilizing passive air sampling devices, modelled using land use regression methodology, including distance to known sources of Mn exposure (61), and enhanced with measures of Mn in community water sources, or sampling settled dust in homes (62).

### Neurotransmitters and brain regions involved

It is critical to gain a better understanding of the neurotransmitter systems and brain regions involved, since there is evidence of the involvement of the cerebellum and brainstem in mutation carriers. Murine models using SLC30A10 and SLC39A14-knockout mice are crucial in understanding the complexities involved (63). A number of neurotransmitter systems implicated in manganese neurotoxicity (30) are of potential diagnostic interest to identify manganism or distinguish it from PD [including dopamine and its metabolites, such as homovanillic acid (HVA) and GABA]. Although HVA levels in cerebral spinal fluid are associated with Parkinsonian symptoms (64), and a previous study that characterized workers chronically exposed to manganese reported associations of HVA and vanillylmandelic acid with serum and urinary manganese (65), human and primate studies have not identified the effects of Mn on dopamine or metabolite concentrations (30).

### Dopamine terminals

A comparison needs to be made between dopamine terminals and release between humans with PD, and those with manganism. In mice and primates, there are normal terminals, but an impaired release with manganism; whereas in subjects with PD, terminals and release are impaired (66).

### Systematic studies on biomarkers

To date, reliable biomarkers of Mn-induced CNS damage have not emerged, at least to the best of our knowledge. Findings derived from experimental animal models have yet to be translated and validated in humans (55), including neurotoxic effects on dopaminergic, GABAergic, glutamatergic, and cholinergic systems. Future approaches should focus on biomarkers that are accurate and reflective of gender, age, and nutritional status. Some general biomarkers detectable in fluidic matrices have been advanced, such as the proteins, S100 calcium binding protein B, neuron-specific enolase, or glial fibrillary acidic protein. Their specificity to Mn-induced CNS damage has yet to be characterized. Additional studies should be directed at the utility of combined screening, i.e., using multiple biomarkers to determine the signature-specific effects of Mn. The successful development of prediction systems and validation requires collaborative efforts across multiple disciplines; if successful however, the results will facilitate the development of risk assessments and management strategies.

### Investigation of the potential of novel biomarkers (metabolomics)

Methods such as metabolomics may hold promise for identifying putative markers of Mn exposure, determining pathways perturbed by Mn exposure and understanding potential early markers of Mn toxicity. There are several limitations and challenges associated with the use of metabolomics for exposure assessment, given the inherent variability in small-molecule metabolites (42). In the most rigorous studies, care will be taken to ensure that samples are taken at the same time of day, and information on relevant covariates such as dietary habits, pre-existing health conditions and the use of pharmaceutical agents should also be collected (43). Given the large number of statistical comparisons made in metabolomics studies, a sufficient number of participants need to be enrolled to ensure that data can be spilt into separate training and testing sets to validate potential findings (42), and appropriate statistical methods for big data need to be utilized. Conclusions drawn from metabolomics studies with adequate sample sizes, that are validated in independent cohorts will ultimately be the most compelling, and as metabolomics gains popularity in exposure assessment and biomarker discovery, standard procedures for the collection and analysis of metabolomics data should be developed and applied to ensure that results are generalizable across different exposed cohorts, and to provide further strength to compelling findings. Despite the challenges of human metabolomics studies, metabolomics holds great promise for understanding subtle biochemical changes associated with Mn exposure, and its potential should be continued to be explored, with care given to the collection, interpretation and reproducibility of the findings (67).

### More MRI data on the accumulation of Mn in workers under different exposure scenarios

Exposure to welding fumes significantly differs from exposure to smelting dust in the size of Mn particulates and the composition of different metals contributing to the exposure, aside from Mn. Such occupational exposure again differs significantly from exposure to Mn through environmental sources. Since Mn may reach the brain through the olfactory nerve bypassing the blood-brain-barrier, it has been suggested that the particulate size, particularly the ratio of ultra-fine or nanosized particulates, may play a crucial role in the amount of Mn reaching the brain at a given total exposure. An understanding of the accumulation of brain Mn with MRI as a function of exposure scenarios, metal mixtures and particulate sizes is still lacking.

### Understanding the role of other metals in the diagnosis of manganism, as low levels of iron are found in cases of hypermanganesemia

In mechanisms of Mn neurotoxicity, mitochondrial dysfunction, oxidative stress and protein aggregation, and alterations in the homeostasis of iron play a crucial role. Both divalent metals Mn and iron act as co-factors for several enzymatic processes in the central nervous system, having similar characteristics and chemical properties. They are transported bound to common cellular transporter proteins (transferrin, ferroportin) and share similar brain distribution (68,69). An imbalance in iron alters the expression of iron transporters that also mediate Mn transport, that affects Mn homeostasis and associated neurochemical functions, modifying Mn transport and associated neurotoxicity. Iron deficiency upregulates transporter proteins that results in increased Mn levels in several tissues including the brain, with an enhanced Mn accumulation prominently in the basal ganglia, and also reducing protective capacity due to decreased cofactor activity for antioxidant enzymes (70).

As iron is a competitive inhibitor of Mn gastrointestinal absorption, iron supplementation therapy may be effective in treating diseases with hypermanganesemia accompanied by low iron and ferritin levels, including disorders with mutations encoding manganese transporter proteins, renal failure undergoing hemodialysis, or chronic iron deficiency (51,68).

## Summary and conclusions

Manganism is a unique movement disorder caused by exposure to high levels of Mn. It can also be a result of genetic or metabolic disorders. Currently, manganism occurs rarely due to reductions in occupational exposure levels associated with modern industrial hygiene practices. Exposure to Mn at lower levels can lead to subtle neurofunctional effects, distinct from manganism following high exposure levels.

Manganism shares clinical features similar to PD, particularly bradykinesia and rigidity. However, manganism is a distinct neurological condition affecting different areas of the brain and is characterized by symptoms that are not present in PD, including atypical tremor, more frequent dystonia and a propensity to fall backwards.

Diagnostic criteria for manganism have been proposed, with the 2005 IRSST (11) criteria being the most recent. These criteria can be used to classify Mn exposure workers into three groups (clinically possible, clinically probable and definite case) based on neurological symptoms, exposure data, diagnostic findings and the exclusion of other neurological diseases. Neurological symptoms include dystonia, bradykinesia, rigidity, postural instability and gait disturbances.

Biomarkers of exposure to and the effects of Mn have been extensively studied in occupational and non-occupational settings. Examined media include blood, plasma, hair, urine, bone, teeth, fingernails and toenails. Studies on biomarkers, however, lack standardized methods for Mn in different media, hindering the comparability of the findings. Although there is currently no strong and consistent evidence for a valid Mn biomarker in any biological medium for clinical purposes, studies on toenails have shown high sensitivity and specificity in discriminating between exposed and unexposed subjects.

Numerous advancements have been made with the use of MRI to assess and quantify Mn accumulation in the brain. In addition to the basal ganglia, considerable Mn accumulation occurs in other parts of the brain, namely the frontal cortex, frontal white matter and the hippocampus. As Mn washes out from parts of the brain following the cessation of exposure, MRI may be limited to characterizing short-term Mn exposure.

Future research activities can lead to the better characterization of manganism. There is a need for a better understanding of the role and timing of Mn deposition in different regions of the brain, neurotransmitter systems and regions involved, dopamine terminals and release between manganism and patients with PD, as well as the role of other metals in the diagnosis of manganism. The identification of potential novel biomarkers may also provide helpful tools for the clinical diagnosis of manganism.

Updates to the relatively dated criteria will allow clinicians to more effectively distinguish between manganism due to Mn neurotoxicity and PD, as well as to take advantage of recent scientific developments in characterizing Mn exposure. Updates to the clinical diagnosis of manganism and Mn neurotoxicity could consider the history of exposure from all sources (occupational and non-occupational excess exposure to Mn, including genetic and metabolic disorders), the presence of clinical signs and symptoms, recent advances in exposure (MRI confirmation of Mn accumulation in the brain after recent exposures) and the pharmacokinetics of Mn. Furthermore, updated criteria may include differential diagnosis of manganism and other neurological diseases with similar symptoms.

## Supplementary Material

Proposed flow diagram for the differential diagnoses of manganism and IPD, based on PET, SPECT and MRI neuroimaging. This accompanies the material presented in Table I in the main manuscript. IPD, idiopathic Parkinson's disease; PET, positron emission tomography; SPECT, single-photon computed tomography; MRI, magnetic resonance imaging. The figure was adapted from the study by Kim (17). Please see the main text for references.

International Workshop on Diagnostic Criteria for Manganism

## Figures and Tables

**Table I:** Similarities and differences between manganism and Parkinson's disease [adapted from the study by Santamaria *et al* (19)].

	Manganism	Parkinson's disease
Clinical features		
Clinical outcomes	Bradykinesia, rigidity, masked face (hypomimia), no tremor^a^, frequent speech disorder^b^, dystonia, falling backward, ‘cock-gait’ walk	Bradykinesia, rigidity, masked face (hypomimia), resting tremor, asymmetry, stooped gait
Pathology	Neuronal loss in basal ganglia, primary target is the globus pallidus; no Lewy bodies^c^	Neuronal loss, primary target is the substantia nigra pars compacta, Lewy bodies observed
Response to treatment	No response to levodopa^d^	Sustained response to levodopa
Neuroimaging outcomes		
PET	Normal F-DOPA PET scan	Reduced F-DOPA uptake in posterior putamen
MRI	Abnormally high signal in the globus pallidus, striatum and substantia nigra	Normal

^a^Modified from ‘tremor’ in original table;

^b^‘frequent speech disorder’ not indicated in original table;

^c^modified from ‘Lewy bodies rare’ in original table;

^d^modified from ‘Initial response to levodopa, but not sustained’ in original table. F-DOPA, fluorodopa; PET, positron emission tomography; MRI, magnetic resonance imaging.

## Data Availability

Not applicable.
