# Supplementary material for: Diagnosis of manganism and manganese neurotoxicity: A workshop report
Source: Med Int (Lond). 2024 Feb 6;4(2):11. doi: 10.3892/mi.2024.135 (PMC10895461; doi:10.3892/mi.2024.135)

Figure S1. Proposed flow diagram for the differential diagnoses of manganism and IPD, based on PET, SPECT and MRI neuro-imaging. This accompanies the material presented in Table I in the main manuscript. IPD, idiopathic Parkinson's disease; PET, positron emission tomography; SPECT, single-photon computed tomography; MRI, magnetic resonance imaging. The figure was adapted from the study by Kim (17). Please see the main text for references.

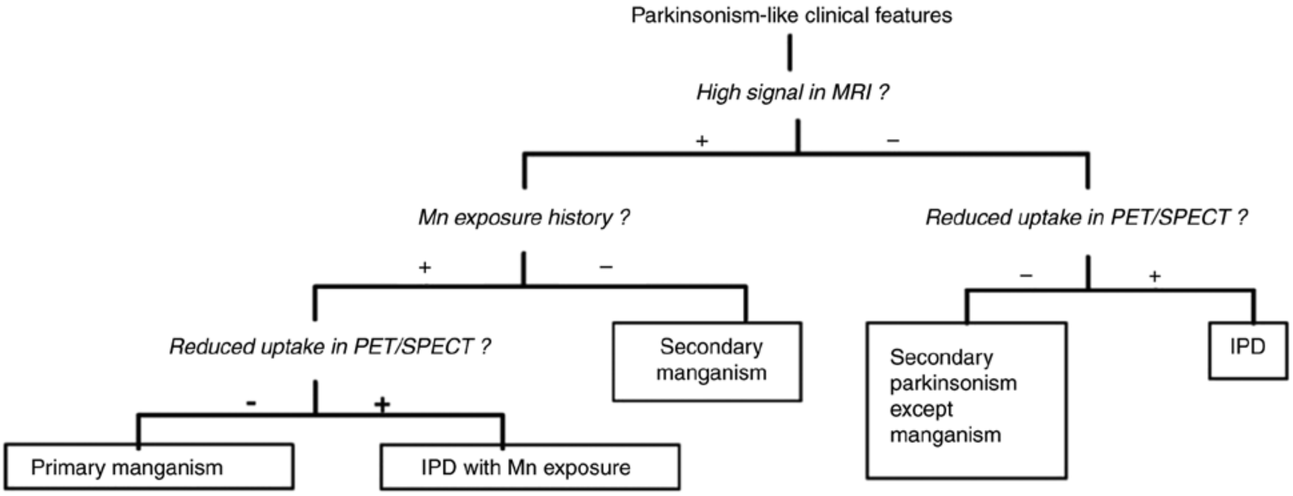

Supplement: Proposed flow diagram for the differential diagnoses of manganism and IPD, based on PET, SPECT and MRI neuroimaging. This accompanies the material presented in Table I in the main manuscript. IPD, idiopathic Parkinson's disease; PET, positron emission tomography; SPECT, single-photon computed tomogr [file Supplementary_Data1.pdf]
