# Supplementary material for: Diagnosis of manganism and manganese neurotoxicity: A workshop report
Source: Med Int (Lond). 2024 Feb 6;4(2):11. doi: 10.3892/mi.2024.135 (PMC10895461; doi:10.3892/mi.2024.135)
Supplement: International Workshop on Diagnostic Criteria for Manganism [file Supplementary_Data2.pdf]

Appendix S1. International Workshop on Diagnostic Criteria for Manganism

November 12-13, 2020

AGENDA

**NOVEMBER 12 – 08:00 to 14:15 (EST)**		
**Introduction and Welcome**		
08:00-08:05	Welcome	Daniel Krewski, University of Ottawa/RSI
08:05-08:30	Introductions	Cemil Alyanak, RSI
08:30-08:40	Meeting Goals	Don Mattison, RSI
08:40-08:55	Current Diagnostic Criteria for Manganism	Franco Momoli, RSI
08:55-09:00	5-minute Health Break	
**Session 1: Background Presentations & Discussions**[1]		
09:00-09:20	Indicators of Neurological Dysfunction	Nataliya Karyakina, RSI
09:20-09:40	PK of Manganese	Siva Ramoju, RSI
09:40-10:00	MRI Markers	Siva Ramoju, RSI
10:00-10:20	Occupational Biomarkers	Nataliya Karyakina, RSI
10:20-10:40	Environmental Biomarkers	Natasha Shilnikova, RSI
10:40-10:45	5-minute Health Break	
**Session 2: Participant Perspectives**		
10:45-10:50	Charge Questions	Don Mattison, RSI
10:50-12:00	Invited Participant Presentations	Participants 1 - 6
12:00-12:30	Meal Break with Informal Discussion	
12:30-13:30	General Discussion	Participants
13:30-14:00	Suggested Key Diagnostic Criteria	Moderator: Don Mattison, RSI<br>Participants

[1] 15-minute presentations followed by 5-minute clarification questions

14:00-14:05	5-minute Health Break	
14:05-14:15	Day's Summary	Don Mattison, RSI
**NOVEMBER 13 – 08:00 to 10:30 (EST)**		
**Session 3: Concluding Discussion**		
08:00-08:05	Welcome	Daniel Krewski, University of Ottawa/RSI
08:05-08:30	Proposed Diagnostic Criteria	Franco Momoli, RSI
08:30-10:00	Discussion of Proposed Criteria	Moderator: Don Mattison, RSI<br>Participants
10:00-10:10	10-minute Health Break	
10:10-10:30	Summary of Workshop	Don Mattison, RSI
